# Chromosome-scale Genome assembly of the critically endangered White-eared Night-Heron (*Gorsachius magnificus*)

**DOI:** 10.1038/s41597-023-02894-6

**Published:** 2024-01-16

**Authors:** Chenqing Zheng, Qing Chen, Shiguo Huang, Weizhen Song, Guoling Chen, Hongzhou Lin, Chunsheng Xu, Xiran Qian, Yachang Cheng, Aiwu Jiang, Zhongyong Fan, Yang Liu

**Affiliations:** 1 State Key Laboratory of Biocontrol, School of Life Sciences, Sun Yat-sen University, 510275 Guangzhou, China; 2 School of Ecology, Shenzhen Campus of Sun Yat-sen University, Shenzhen, 518107 China; 3 The Forestry Bureau of Chun’an County, Chun’an, 510275 Zheijang China; 4 School of Biological Sciences, The University of Hong Kong, Hong Kong, China; 5 Zhejiang Museum of Natural History, Zhejiang Biodiversity Research Center, Hangzhou, 310014 China; 6 Guangxi Key Laboratory of Forest Ecology and Conservation, College of Forestry, Guangxi University, Nanning, 530004 China

**Keywords:** Genome, Evolutionary genetics

## Abstract

The White-eared Night-Heron (*Gorsachius magnificus*, *G. magnificus*) is a critically endangered heron that is very poorly known and only found in southern China and northern Vietnam, with an estimated population of 250 to 999 mature individuals. However, the lack of a reference genome has hindered the implementation of conservation management efforts. In this study, we present the first high-quality chromosome-scale reference genome, which was assembled by integrating PacBio long-reads sequencing, Illumina paired-end sequencing, and Hi-C technology. The genome has a total length of 1.176 Gb, with a scaffold N50 of 84.77 Mb and a contig N50 of 18.46 Mb. Utilizing Hi-C data, we anchored 99.89% of the scaffold sequences onto 29 pairs of chromosomes. Additionally, we identified 18,062 protein-coding genes in the genome, with 95.00% of which were functionally annotated. Notably, BUSCO assessment confirmed the presence of 97.2% of highly conserved Aves genes within the genome. This chromosome-level genome assembly and annotation will be valuable for future investigating the *G. magnificus*’s evolutionary adaptation and conservation.

## Background & Summary

The White-eared Night-Heron *G. magnificus* is a nocturnal wader bird that is mainly distributed in southern China and northern Vietnam^1,2^. It belongs to the Ardeidae family and inhabits dense forests with abundant-watered areas, marshes, and reservoirs^3^. This species is listed as an Endangered species on the IUCN Red List^4^ with an estimated population of 250–999 mature individuals^1^ and is poorly known due to its rarity and few localities in the wild^4,5^. The increasing demands of humans for timber and agricultural land, intensive use of agricultural chemicals, and hunting are the main threats to its survival. It is urgent and challenging to evaluate the genetic status of *G. magnificus* due to conservation status and scattered localities^2^. Mitochondrial phylogenetic relationships suggest *G. magnificus* is not closely related to other members of *Gorsachius* but might be more closely related to heron species^6^. A recent phylogeny robust of herons based on ultraconserved elements revealed this species is a sister species of the African-distributed White-backed Night Heron (*Gorsachius leuconotus*)^7^. However the purpose of assembling a chromosome-level genome of an endangered night heron species has three advantages: 1) to be a high-quality reference genome for other population genomic studies in the family Ardeidae; 2) to allow comparative genomic studies of nocturnal birds to reveal local adaptation; 3) to carry out conservation genomics of this endangered species. Therefore, the availability of this genome facilitates tackling some challenges in evolution, conservation, and ecological studies^8^.

In this research, we have successfully generated a high-quality reference genome of *G. magnificus* at the chromosomal level, employing a comprehensive approach that integrates PacBio long-read sequencing, chromosome conformation capture (Hi-C) technology, and Illumina platform paired-end short-read sequencing. The assembled genome spanned a total length of 1.176 Gb, organized into 539 contigs and 79 scaffolds. The contig N50 length reached 18.46 Mb, while the scaffold N50 length was 84.77 Mb. Subsequently, 29 pairs of chromosomes with a total of length 1.175 Gb were anchored utilizing Hi-C technology, which corresponds to 99.89% of the assembled sequences. Moreover, we have identified 18,082 protein-coding genes based on *de novo* and homolog-based strategies, and 95% of these genes (17,177) were functionally annotated in publicly available databases including Gene Ontology, KEGG, and Pfam. Additionally, a BUSCO analysis demonstrated the completeness of 97.2% of annotated genes. This high-quality genome not only offers a reference genome for conservation genomics of *G. magnificus* but also facilitates phylogenomic and comparative genomic studies on a relatively understudied avian family, Ardeidae.

## Methods

### Ethics statement

Sample collection for scientific research purposes was in accordance with the ethical conditions in the Chinese Animal Welfare Act (20090606) and was approved by Forestry Administration of Guangdong Province, China (DFGP Project of Fauna of Guangdong-202115).

### Sampling, DNA/RNA extraction, library construction, and sequencing

In our study, we gathered samples from a dead male *G. magnificus* specimen, which had resided at a wildlife rescue center in Shandong, Guangdong, China. In the genomic assembly of bird species, sampling female individuals offers an opportunity to obtain the W chromosome. However, we did not achieve this ideal condition for such an endangered and cryptic species. Genomic DNA was extracted from muscle tissue and utilized for whole genome sequencing and subsequent *de novo* assembly. Additionally, we obtained a total of nine RNA samples from various tissues within the same male individual, including the brain, lung, testis, thigh muscle, liver, pectoralis muscles, wing muscle, cardiac muscle, and eyeball, for RNA sequencing (RNA-seq) analysis.

We extracted high-molecular-weight genomic DNA from muscle samples following the instructions of CTAB (Cetyl trimethyl ammonium bromide), specifically for the purpose of *de novo* genome sequencing. We assessed the integrity and quality of the genomic DNA through agarose gel electrophoresis and a Qubit Fluorometer. For genome survey and polishing, we sequenced a single shotgun library with a 350-bp insert size on the Illumina NovaSeq 6000, yielding approximately 35.79 Gb (equivalent to a 30.43X coverage) of 150-bp paired-end reads (see Table 1). To facilitate genome assembly, SMRTbell libraries were created with an average 20-kb insert size using the SMRTbell Template Prep Kit 1.0 (Pacific Biosciences). Subsequently, we employed Blue Pippin (Labgene Scientific) to select fragment sizes, and we conducted library sequencing using the PacBio platform, utilizing single molecule real-time (SMRT) sequencing (PacBio RSII) technology, which generated a total of 128.52 Gb (equivalent to a 109.29X coverage) of data (see Table 1). For Hi-C sequencing, we fixed muscle tissue from the same male individual intended for *de novo* genome sequencing with 37% formaldehyde. Following a 10-minute incubation at room temperature, we halted the cross-linking reaction with 2.5% formaldehyde. We then collected the precipitated cells for Hi-C library preparation. A single Hi-C library was constructed and subjected to paired-end sequencing with 150 bp reads on the Illumina NovaSeq 6000 Sequencing System, resulting in a total of 110.6 Gb (94.05X coverage) of data (see Table 1). For RNA sequencing, we extracted total RNA from nine different tissues using the RiboPure^TM^ RNA Purification Kit (Ambion^®^) and assessed its integrity with the RNA Nano 6000 Assay Kit on the Bioanalyzer 5400 system (Agilent Technologies, CA, USA). Following the manufacturer’s instructions, we constructed RNA libraries. All libraries were subjected to 150-bp paired-end sequencing on the Illumina NovaSeq 6000, and after adapter trimming and quality filtering using fastp (v 0.23.2)^9^ with default parameters, we obtained a total of 57.6 Gb of high-quality RNA sequencing data (see Table 2).

**Table 1:** Summary of sequencing strategy.

Sequencing Strategy	Sequencing platform	Library size	Total data (Gb)	Sequence coverage (X)
PacBio	PacBio RSII	20kb	128.52	109.29
Illumina	Illumina NovaSeq PE150	350bp	35.79	30.43
Hi-C	Illumina NovaSeq PE150	350bp	110.6	94.05
Total	—	—	274.91	233.77

**Table 2:** Summary statistical of nine tissue’s transcriptome sequencing data.

Sample	Raw Reads	Raw Base (Gb)	Clean Reads	Clean Base (Gb)	Q20 (%)	Q30 (%)	GC content (%)
Testis	44167220	6.63	43657536	6.43	97.39	93.0	50.41
Lung	45508560	6.83	45140894	6.65	97.51	93.12	47.89
Eyeball	46043764	6.91	45632468	6.73	97.22	92.52	48.11
Brain	40197324	6.03	39898874	5.86	97.47	93.01	45.88
Pecloralis muscles	43775816	6.57	43201702	6.4	97.16	92.54	51.16
Wing muscle	45449080	6.82	44899000	6.67	97.2	92.6	51.05
Thigh muscle	45066818	6.76	44446696	6.56	97.3	92.88	51.57
Cardiac muscle	44627758	6.69	44151946	6.5	97.31	92.81	49.58
Liver	39781586	5.97	39165872	5.8	97.34	92.97	50.77

### Genome assembly

**Fig. 1:**
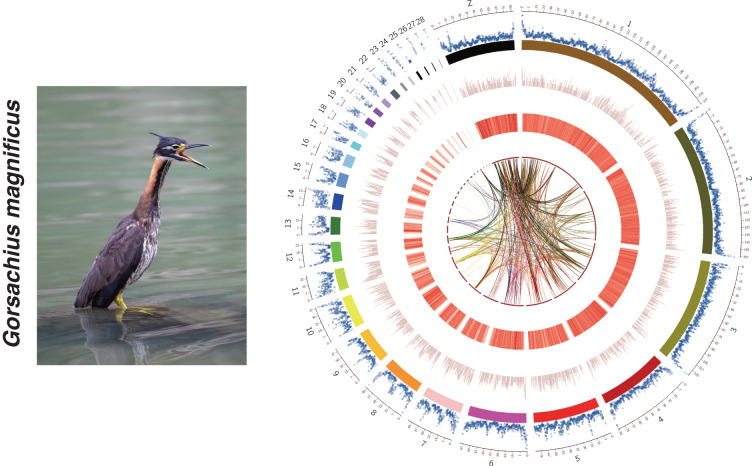
Characterization of assembled *Gorsachius magnificus* genome. From inner to outer layers for circle figure (right): Collinearity of different chromosomes, Distribution of SNPs, Genes abundance, Chromosomes, Density of GC content. (Left figure provided by Liao Zhikai).

**Fig. 2:**
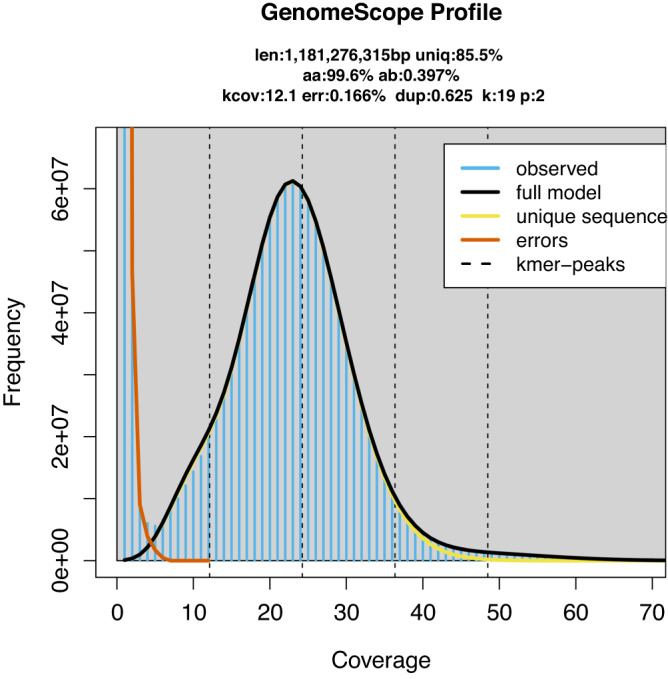
K-mer frequency and genome size evaluation of *Gorsachius magnificus* genome.

The chromosome-scale reference genome was assembled by combining PacBio long reads, Illumina short reads, and Hi-C sequencing data. Firstly, We obtained a genome size estimation of 1,181.27Mb and a heterozygosity rate of 0.397% by jellyfish (v 2.3.0)^10^ and GenomeScope (v 2.0.0)^11^ using Illumina short reads (Fig. 2). Then, we generated the initial draft contig assemblies based on high-coverage PacBio long reads data using wtdbg2 (v 2.5) and wtpoa-cns (v 1.1)^12^, the draft genome of the *G. magnificus* contained 539 contigs with a contig N50 of 18.46 Mb and a total length of 1.176 Gb (Table 3). Subsequently, PacBio long-read and additional Illumina paired-end short reads were applied to polish the draft genome following the wtdbg2 pipeline. To join contigs into scaffolds, 3D-DNA (v 190716)^13^ was used to produce the initial chromosomal results by aided by Hi-C data. These scaffolds were roughly reviewed and adjusted using Juicebox (v 1.11.08)^14^ and then further polishing the assembly using 3D-DNA. Finally, we obtained the chromosome-level assembly genome consisting of 79 scaffolds with an N50 of 84.77 Mb, and 99.89% genome was reordered and anchored onto 29 pairs of chromosomes (Table 3), with their lengths ranging from 60.39 kb to 218.43 Mb (Fig. 1, Table 4). The GC contents of the final assembled genome were 42.88% (Fig. 1, Table 3).

**Table 3:** Summary of *Gorsachius magnificus* genome assembly and annotation.

Genomic Resource	value
**Draft genome assembly**	
Total Size (bp)	1,176,285,410
No. of contig	539
Mean length (bp)	2,182,347
N50 (bp)/rank	18,463,888/19
N90 (bp)/rank	4,115,765/65
Max contig size (bp)	62,038,978
Min contig size (bp)	2099
**Chromosome-length assembly**	
Total Size (bp)	1,176,607,910
No. of scaffold	79
Mean length (bp)	14,893,771
N50 (bp) /rank	84,772,300/3
N90 (bp) /rank	21,490,085/13
Max scaffold size (bp)	218,435,882
Min scaffold size (bp)	2,099
BUSCO completeness	Complete:96.6% [S:95.9%, D:0.4%], Fragmented:0.7%, Missing:2.7%
**Genome characteristics**	
GC content	42.88%
No. of predicted protein-coding gene	18062
No. of transcripts with some UTR annotation	17676 (97%)
Average transcript length (Kb)	22.8
Average CDS length (Kb)	1.6
Average exon length (bp)	166.3
Average number of exon	9.7
**Repetitive sequences (% of genome)**	
SINEs (Mb)	1.3 (0.11%)
LINEs (Mb)	29.4 (2.5%)
LTR (Mb)	64.1(5.45%)
Total (Mb)	122.4 (10.41%)
**Genome annotation**	
No. of functionally annotated protein-coding gene	17177 (95%)
No. of genes with GO annotation	14600 (80%)
No. of genes with KEGG annotation	12754 (71%)
BUSCO completeness	Complete:97.2% [S:83.9%, D:13.3%], Fragmented:0.9%, Missing:1.9%

CDS: coding region sequences; LINE: long interspersed nuclear elements; SINE: short interspersed nuclear elements; LTR: long terminal repeat; BUSCO: S, single-copy; D, duplicated BUSCOs.

**Table 4:** Summary of chromosome length of *Gorsachius magnificus* genome.

Chromosome	Length (bp)	Percentage (%)
chr1	218435882	18.56
chr2	158848200	13.50
chr3	126472151	10.75
chr4	82945395	7.05
chr5	80596957	6.85
chr6	70974105	6.03
chr7	49616730	4.22
chr8	47714487	4.06
chr9	40831957	3.47
chr10	38875770	3.30
chr11	25946990	2.21
chr12	24012853	2.04
chr13	21490085	1.83
chr14	19429904	1.65
chr15	16712334	1.42
chr16	14722482	1.25
chr17	8180870	0.70
chr18	7697788	0.65
chr19	7586621	0.64
chr20	6208117	0.53
chr21	6051309	0.51
chr22	6693957	0.57
chr23	3159198	0.27
chr24	2688418	0.23
chr25	2588482	0.22
chr26	1106936	0.09
chr27	604855	0.05
chr28	60392	0.01
chrZ	84772300	7.20
Total	1175025525	99.87
Unplaced	1582385	0.13

To assess the quality of genome assembly and annotation, we used BUSCO (v 5) (Benchmarking Universal Single-Copy Orthologs)^15^ with aves_odb10 contains 8,338 single-copy orthologs as a reference to evaluate the completeness of the genome, the BUSCO research for assessment of the genome completeness showed that 96.6% of the BUSCO genes were complete, 0.7% were fragmented, and 2.7% were missing (Table 3).

### Repeat sequences and genome annotation

To annotate the genome of the *G. magnificus*, we identified 122.4 Mb of repetitive sequences, accounting for 10.41% of the genome by a combination of homology-based and *de novo*-based identification, manual curation, and classification^16^ (Table 3). Repeatmasker (v 4.1.2)^17^ was used to search homology sequence from the Repbase library (v 20181026) for Aves, and *de novo* prediction was performed using Repeatmodeler (v 2.0.8)^18^, we furthermore used an additional method, EDTA (v 2.0.1)^19^, to annotate LTRs. This method combines the raw predictions of LTRharvest (v 1.1)^20^, LTR_FINDER_parallel (v 1.2)^21^, and LTR_retriever (v 2.9)^22^.

Next, we utilized three methods to predict protein-coding genes: transcriptome-based prediction, homology-based prediction, and *de novo* prediction. First, we assembled the transcriptome data from different tissues using Trinity (v 2.13.2)^23^. The homologous gene sets were obtained from the protein and transcript sequences of 15 proximate bird genomes (Table S1). Then, we performed Maker (v 3.01.03)^24^ for *de novo* prediction, and GeMoMa (v 1.7.1)^25^ for homology-based prediction to identify the protein-coding genes. Finally, we generated non-redundant gene sets from the three data sets. We also used GeMoMa to predict the UTR regions of protein-coding genes based on transcriptome sequences.

The functional annotation of protein-coding genes was constructed by mapping gene sets to protein databases Gene Ontology (GO)^26^ and Kyoto Encyclopedia of Genes and Genomes (KEGG)^27^ using eggNOG-mapper^28^.

We also used the combination of *de novo*, homolog-based, and transcript-based methods, 18,062 protein-coding genes (Genes) were predicted, and 97% of predicted regions with UTR region. A total of 17,177 (95% of Genes) were successfully annotated with at least one function term by searching against functional databases (Gene Ontology, KEGG, Pfam). we also used BUSCO (v 5) to evaluate the completeness of the annotation, The analysis for assessment of annotation completeness revealed a complete recall of 97.2% (83.9% single-copy; 13.3% duplicated) of genes, 0.9% fragmented, and 1.2% missing (Table 3).

## Data Records

The Raw data of PacBio are deposited into NCBI SRA with accession number SRR26858085, and the Illumina WGS, Hi-C, and RNA-seq sequencing data were stored under accession numbers SRX22552595- SRX22552607^29^. The genome assembly has been deposited in the GeneBank database under the accession number JAXBDB000000000^30^. The genome annotations are available from the Figshare repository^31^.

## Technical Validation

**Fig. 3:**
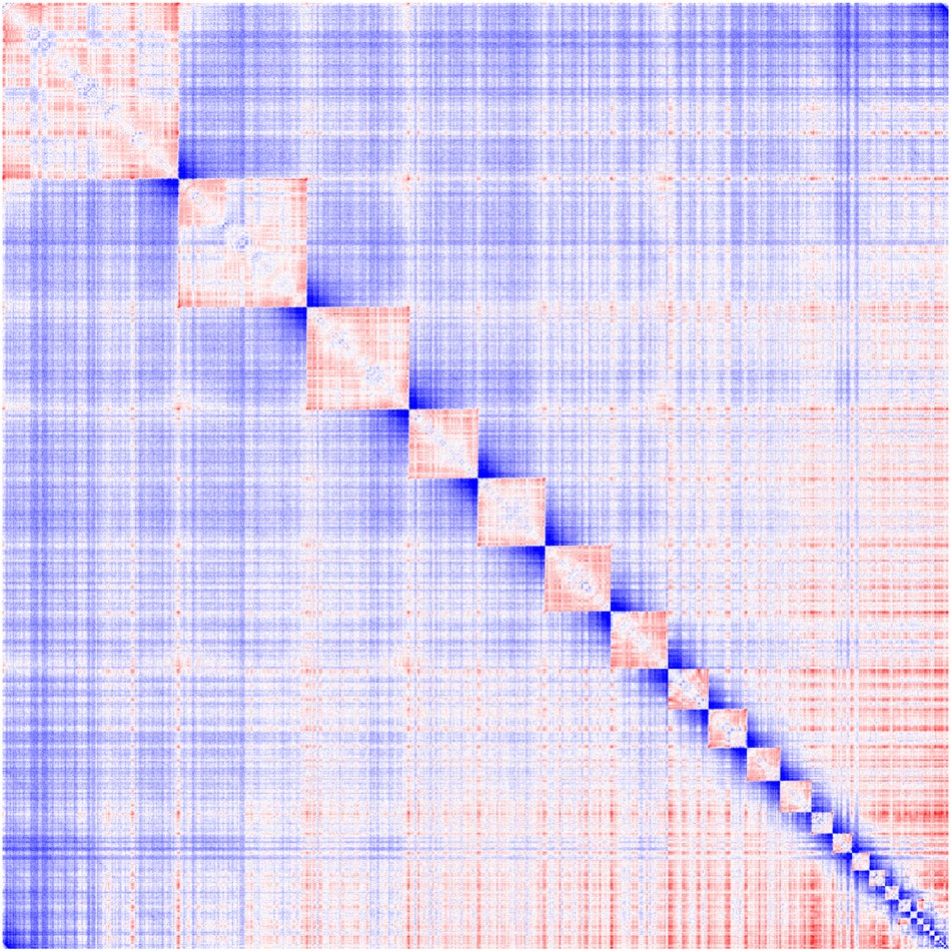
Hi-C interactive heatmap of genome-wide of *Gorsachius magnificus*. The coordinates in the figure indicate genome length. The deeper red means a stronger interaction between the genomics regions.

The assembled genome of has a size of 1.176 Gb, and its scaffold N50 is 84.77 Mb. This is very close to the estimated size of 1.181 Gb from our kmer-analysis (Fig. 2). The Hi-C heatmap displays a well-organized interaction pattern within the chromosomal regions (Fig. 3). Notably, 99.89% of the genome bases have been anchored onto 29 pairs of chromosomes (Figs. 1, 3, Table 4). The genome assembly quality assessment shows 96.6% completeness using BUSCO, with the protein-coding sequences achieving 97.2% completeness (Table 3).

**Fig. 4:**
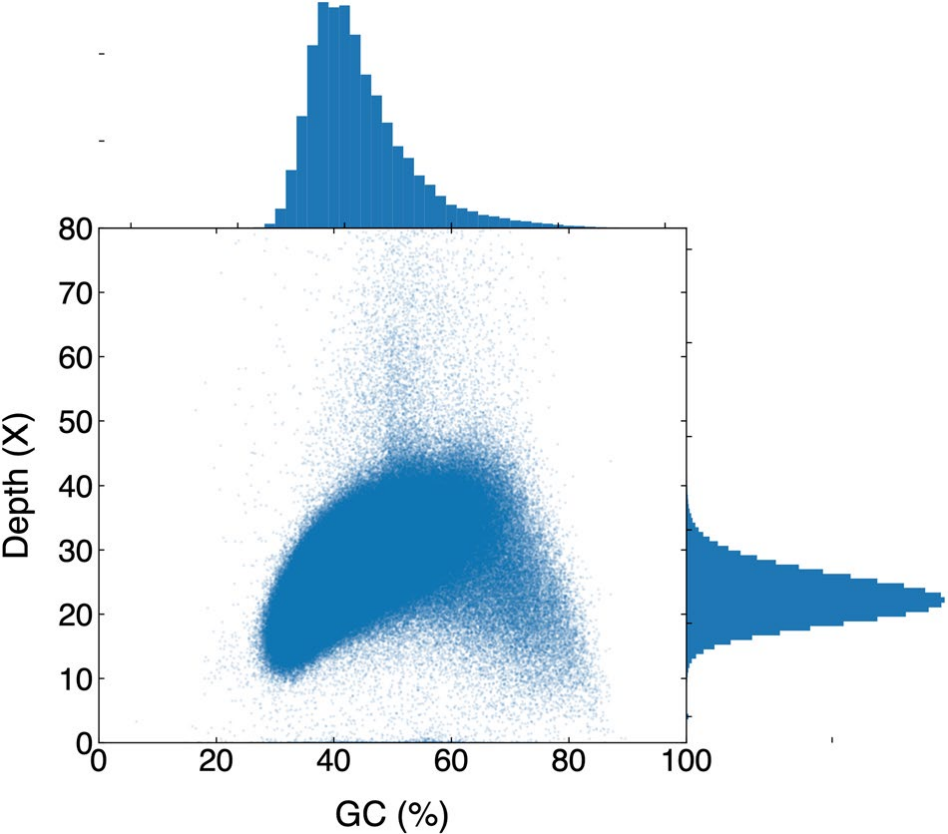
The average sequencing depth and the distribution of GC content of *Gorsachius magnificus*. The specific GC content and sequencing depth were calculated in each window with a 1000bp size, corresponding to a dot in the figure. The Y-axis and left histogram represent the average sequencing depth, the peak value of the left histogram is about 25. The X-axis and top histogram distribution represent the GC content, the peak value of the top histogram is about 0.4.

We used the sequence alignment method to evaluate possible contamination and the completeness of the genome assembly. The Burrows-Wheeler Aligner (BWA, v 0.7.6)^32^ was used to map Illumina short reads to the assembled genome with default parameters. Importantly, the relationship between sequencing depth and GC content distribution revealed no deviations from the expected levels, allaying concerns about contamination or sequencing biases (Fig. 4). Additionally, our mapping results indicate that 99.03% of reads were successfully mapped, and the coverage rate was approximately 99.77% (Table 5), confirming the alignment consistency between the reads and the assembled genome. Compared to other assembled avian genomes, the main measures like the scaffolds N50 and the number of genes are very close to *G. gallus* and a recently published chromosome-level genome *A. baeri)* (Table 6).

**Table 5:** Statistical results of Illumina short-reads alignment.

Items	Value
Mapping rate (%)	99.03
Average sequencing depth (X)	29.79
Coverage of genome >= 1X (%)	99.77
Coverage of genome >= 4X (%)	99.65
Coverage of genome >= 10X (%)	98.58
Coverage of genome >= 20X (%)	78.15

**Table 6:** Comparative analysis of other assembled avian genomes.

Species	Genome length (Gb)	No. chromosomes	No. Scaffolds	Scaffolds N50 (M)	No. Genes	GC content(%)
*G.magnificus*	1.18	29	79	84.8	18,062	42.88
*N.nycticorax*^33^	1.18	—	7,611	3	13,361	42.5
*G.gallus*^34^	1.05	41	213	90.9	18,023	42
*A.baeri*^35^	1.14	35	135	85.8	18,581	41.94

### Supplementary information

Supplementary Information

## Data Availability

**Genome assembly:** 1. jellyfish: parameters: -m 19 -s 100M 2. GenomeScope: all parameters were set as default 3. wtdbg2: parameters: -x sq -g 1G -K 2000 –edge-min 4 -p 19 -S 4 -L 5000 –tidy-reads 8000 4. wtpoa-cns: all parameters were set as default 5. 3D-DNA: all parameters were set as default 6. Juicebox: all parameters were set as default 7. BUSCO: parameters: -l busco_downloads/lineages/aves_odb10 **Genome annotation:** 1. RepeatMasker: parameters: -poly -xsmall -engine ncbi -no_is 2. Repeatmodele: parameters: -engine ncbi 3. EDTA: parameters: –species others –step all –anno 1 -t 30 –rmout RepeatMasker.out 4. Trinity: parameters: –seqType fq –SS_lib_type RF –normalize_reads 5. Maker: all parameters were set as default 6. GeMoMa: GeMoMaPipeline parameters: GeMoMa.p = 20 GeMoMa.c = 0.3 AnnotationFinalizer.r = NO; AnnotationFinalizer parameters: u = YES; 7. eggNOG-mapper: all parameters were set as default **Whole genome alignment:** 1. BWA: all parameters were set as default The parameters not mentioned analysis modules in our study were used as default parameters.
